# Effect of the New Plant Growth Biostimulants Based on Amino Acids on Yield and Grain Quality of Winter Wheat

**DOI:** 10.3390/molecules23020470

**Published:** 2018-02-21

**Authors:** Małgorzata Popko, Izabela Michalak, Radosław Wilk, Mateusz Gramza, Katarzyna Chojnacka, Henryk Górecki

**Affiliations:** 1 Department of Advanced Material Technologies, Faculty of Chemistry, Wrocław University of Science and Technology, Smoluchowskiego 25, 50-372 Wrocław, Poland; malgorzata.popko@pwr.edu.pl (M.P.); radoslaw.wilk@pwr.edu.pl (R.W.); katarzyna.chojnacka@pwr.edu.pl (K.C.); henryk.gorecki@pwr.edu.pl (H.G.); 2 AGRECO Ltd., Wrocław, al. Lipowa 21/1, 53-124 Wrocław, Poland; m.gramza@agreco.pl

**Keywords:** plant growth biostimulants, amino acids, winter wheat, field experiments, grain quality

## Abstract

Field and laboratory experiments were carried out in 2012–2013, aimed at evaluating the influence of new products stimulating plant growth based on amino acids on crop yield, characteristics of grain and content of macro- and micronutrients in winter wheat (*Triticum aestivum* L.). The tests included two formulations produced in cooperation with INTERMAG Co. (Olkusz, Poland)—AminoPrim and AminoHort, containing 15% and 20% amino acids, respectively, and 0.27% and 2.1% microelements, respectively. Field experiments showed that the application of products based on amino acids influenced the increase of grain yield of winter wheat (5.4% and 11%, respectively, for the application of AminoPrim at a dose 1.0 L/ha and AminoHort at dose 1.25 L/ha) when compared to the control group without biostimulant. Laboratory tests showed an increase of technological characteristics of grain such as ash content, Zeleny sedimentation index and content of protein. The use of the tested preparations at different doses also contributed to the increase of the nutrients content in grains, in particular copper (ranging 31–50%), as well as sodium (35–43%), calcium (4.3–7.9%) and molybdenum (3.9–16%). Biostimulants based on amino acids, tested in the present study, can be recommended for an efficient agricultural production.

## 1. Introduction

In modern agriculture, along with fungicides, herbicides and insecticides, various products classified as plant growth stimulants are used [1,2]. This relatively new group of products is used to maximize crop yield and quality, especially under unfavorable for plant growth and development environmental conditions [3,4,5]. The role of biostimulants is to control and accelerate the life processes of plants, increase the resistance to stress and stimulate their development (roots and leaves) [1,3]. Biostimulants contribute to better seed germination and induce biological activity of plants. These products are also safe for the environment and contribute to sustainable, high-output low-input crop productions [3,4]. Their application helps to reduce the amount of chemicals used in agriculture and plant protection [4]. One type of biostimulants are preparations based on amino acids [5,6,7,8,9,10]. Products on the market containing amino acids include: Metalosate Calcium and Metalosate Fe (Albion Minerals, Layton, UT, USA); Agrocean B (Agrimer, Plouguerneau, France); Tecamin Brix, Tecamin Max, Tecnokel Amino Mix, and Terra-Sorb Foliar (Agritecno Fertilizantes, Valencia, Spain); Amino Quelant Ca (Bioibérica, Barcelona, Spain); Bosfoliar Activ (COMPO EXPERT, Münster, Germany); NaturalCrop SL (NaturalCrop Poland Sp. z o.o., Warszaw, Poland); and Delfan Plus (Tradecorp, Madrid, Spain).

Amino acids for the production of biostimulants are obtained by chemical synthesis, from plant proteins (e.g., algae, corn, and soybean), as well as from animal proteins by chemical or enzymatic hydrolysis [1,11,12,13]. Amino acids are the basic building blocks of proteins and fulfill multiple functions in the plant—structural, metabolic and transport (Table 1) [14].

Plants can produce amino acids, but this synthesis is highly energy consuming. Therefore, the application of ready for uptake amino acids allows plants to save energy and increase the pace of their development or reconstruction, especially in critical times of plant development [12,14,15,16,17]. Amino acids are also known in the industry of products for agriculture as chelates of metal ions. Microelements chelated with amino acids form very small, electrically neutral molecules, which accelerate their absorption and transport within the plant [15,18,19]. These types of products are beneficial for plants that require supplementation with microelements. For example, the cultivation of high quality winter wheat varieties needs an adequate supply of micronutrients, which act as stimulants for macronutrients, in particular nitrogen [20]. The sensitivity of winter wheat to the micronutrient deficiencies in soil, mainly copper and manganese, is higher than in other crops [20,21,22]. The availability of micronutrients is limited by the characteristics of the soil (pH and sorption capacity), as well as the agricultural practices (fertilization system without plowing, and intensification of crop production and consequently uptake of nutrients). Therefore, foliar application of micronutrients seems to be more effective than soil and it is becoming more popular [20,23,24].

The aim of the present study was to determine the effect of foliar application of new preparations based on amino acids on the yield and grain quality of winter wheat. These are the preliminary studies on the properties and the effectiveness of hydrolysates obtained from feathers. These two hydrolysates differed in the content of amino acids (15% vs. 20%) and elements (17% vs. 23%). The aim of this study was also to check the difference in the uptake of microelements by plants in the presence of different doses of amino acids, i.e., whether the presence of amino acids affects the bioavailability of microelements. This enabled assessing the application rate of the product. Field trials will continue for several years to register a new product and to implement it to the market.

## 2. Results and Discussion

In the present study, we tested the effect of new products based on amino acids produced by chemical hydrolysis of feathers—AminoPrim (at a dose 1.0 L/ha) and AminoHort (at doses 1.0 and 1.25 L/ha) —on the growth, yield and the grain quality of winter wheat. The chemical composition of the preparations based on amino acid is given in Table 2. AminoPrim contains 15% amino acids and 17% elements—16.5% of macroelements and 0.27% of microelements. The second product has higher contents of both amino acids (20%), and macro- (20.5%) as well as microelements (2.1%).

The obtained products, as well as a reference biostimulant of plant growth—Asahi SL—were tested in the field trials without additional fertilization to simulate the abiotic stress and to access the biostimulant properties of the amino acids based products. In the field conditions, plants from all tested groups were exposed to the same abiotic stress. We wanted to compare the biostimulant effect on plants of AminoPrim and AminoHort, as well as in relation to the commercial biostimulant, and a control group without the application of any bioactive substances. In future experiments, we plan to conduct these experiments with standard fertilization to increase the wheat yield. Deficiency of fertilizer nutrients is the form of abiotic stress. The role of plant growth biostimulants is to diminish the effect of this stress on plants growth. We wanted to investigate how efficient are elaborated products in coping with the effects of this stress. This justified the applied methodology.

The results of the field trials showed that the plant vigor was similar for the used preparations based on amino acids (AminoPrim and AminoHort), the reference biostimulant and for the untreated plants without biostimulant (5.0 optimum vigor). Leaf color of plants for all the treatments was also the same as for the control group (5.0 optimal color). Additionally, uneven ears ripening appeared on the tested plots. It was probably because the standard fertilization was not applied. During the experiments, no phytotoxic symptoms were observed on the winter wheat for both products based on amino acids and the commercial biostimulant (no phytotoxicity). On all plots, no lodging of plants occurred. The average crop height on the tested and reference plots was similar when compared to the crop height on the untreated plots (Table 3). There were no statistically significant differences between the groups.

According to the literature data, tillering, nutrient accumulation and yield of winter wheat are influenced by nitrogen form [25]. The survival of tillers influences final ear number which mainly influences yield. In the present study, the tillering evaluation (separately for the first, second and third tiller in the tested groups) did not demonstrate any significant differences in the tillers number (first, second and third tiller) between all the tested products and the control group (Table 4). However, the biggest differences were observed for the third tiller. The average tillers number in the group with AminoHort was 75% higher than in the control group; for AminoPrim 50% higher; and for Asahi 25% higher. This could result from the fact that, among all tested products, AminoHort contained the highest amount of amino acids (20%).

The number of grains per ear did not significantly differ between the tested combinations. The positive effect of the application of plant growth stimulants based on amino acids on the number of ears per m^2^ when compared to the control group was observed: for AminoHort at a dose 1.0 L/ha, it was 13% higher; for AminoHort 1.25 L/ha, 4.5% higher; and for AminoPrim 1.0 L/ha, 4% higher. The best results were obtained for the reference product—ear number per m^2^ was by 17% higher than in the control group (Table 5). In Table 5, we present the average grain yield of the winter wheat. For all the experimental objects, statistically significant differences were not found. The grain yield (t/ha) of winter wheat increased especially in the group with AminoHort at a dose 1.25 L/ha (11%) when compared with the control group. In the group with AminoPrim and Asahi SL, the yield was slightly higher than in the control group (5.4% and 2.0%, respectively). Only for AminoHort at dose 1.0 L/ha, the yield was lower than in the control group. However, it could be caused by relatively high standard deviation obtained in this group. The small differences in the grain yield between the tested groups and the control can result from the application time of the tested preparations. Probably during their application, there was no abiotic stress or the plants were provided with sufficient amounts of nutrients from the environment. It is also supposed that the dose of AminoHort should be higher than 1.0 L/ha, since, at this dose, differences were not observed. Further studies with higher doses of the biostimulant preparations will be undertaken.

Subbarao et al. [8] also observed a positive effect of protein hydrolysate (used in soil and foliar treatments) on plant growth and yield of crops (rice, finger millet, radish and cowpea). It was found that soil application was more effective than foliar application. The application of protein hydrolysate increased root and shoot growth, as well as leaf area [8]. Similar results were obtained in the work of Colla et al. [5], who tested the effect of plant-derived protein hydrolysate containing amino acids and small peptides on the hormone-like activity, nitrogen uptake and growth stimulation of corn, tomato and dwarf pea. It was found that this hydrolysate elicited a hormone-like activity (auxin- and gibberellin-like activity), enhanced nitrogen uptake (by the extensive root apparatus and the increase of nitrogen assimilation process) and consequently crop performance [5]. In the work of Ertani et al. [6], it was found that protein hydrolysates obtained from plants—alfalfa—and from meat flour revealed a different degree of hydrolysis (29% alfalfa and 18% meat flour) and a different amino acid composition (free α-amino nitrogen: 5.6 mg/g alfalfa and 12.5 mg/g meat flour). Nevertheless, both hydrolysates increased root and leaf growth [6].

The analysis of the wheat grains has shown no statistically significant differences for most of the physicochemical parameters, except for the ash content (Table 6). The ash content in grains was higher by 5.6%, 7.8% and 7.2%, respectively, for AminoPrim (1.0 L/ha) and AminoHort at doses of 1.0 and 1.25 L/ha when compared to the control group. The tested products AminoPrim and AminoHort are a source of not only amino acids, 15% and 20%, respectively, but also micro- and macroelements. AminoPrim is a typical amino acid plant growth biostimulant with the total amount of macroelements (N, P, K, Ca, Mg, and S) of 16.5% and the small content of microelements (B, Cu, Fe, Mn, Mo, and Zn) of 0.27%. In the case of AminoHort, these values are as follows: macroelements 20.5% and microelements 2.1%. This biostimulant can also supply cultivated plants (beside ready building blocks, i.e., amino acids) with elements in the case of their critical deficiencies. Amino acids are known to facilitate the transport of elements (metal translocation through xylem) [26]. Therefore, it is supposed that, in the present study, the biofortification of wheat grain occurred. The ash content is a measure of the mineral content of the grain, which is crucial from the nutritional point of view [27]. The highest content of ash was for grains treated with AminoHort at a dose 1.0 L/ha.

For the other technological characteristics of the winter wheat grain, such as Zeleny sedimentation index (indicates baking properties), protein, starch, and wet gluten content, significant increases were not observed in the groups with new biostimulants. The largest value of Zeleny sedimentation index was observed in the case of the application of AminoHort: at a dose 1.25 L/ha, an increase of 9.7% when compared to the control. For AminoHort at a dose 1.0 L/ha and AminoPrim, increases of 9.1% and 2.4%, respectively, were found. Zeleny value was determined by measuring the rate of sedimentation of wheat flour suspended in an acid solution. A high Zeleny value (sedimentation volume) is associated with a high protein content and good baking quality [28]. In the present study, grains from the group treated with AminoHort had the highest protein content and, consequently the highest Zeleny sedimentation index.

The protein content in the wheat grains was also the highest when AminoHort was used at a dose of 1.25 L/ha, i.e., an increase of 3.1%, while the application of AminoHort and AminoPrim at 1.0 L/ha both resulted in increases of 2.3%. When compared to the commercial biostimulant, the content of protein increased on average by 1.8%. AminoHort was a richer source of N_total_ (3.5%) when compared with AminoPrim (2.8%). This coincides with the literature data: more protein content is found in grains fertilized with a higher dose of nitrogen when compared to grains fertilized with a standard dose. The high nitrogen dose can also increase the amount of gluten in grains [29]. In the present study, the content of wet gluten in the grains was ~2% higher than in the control group for AminoPrim (1.0 L/ha) and AminoHort (1.25 L/ha), and ~5% higher than in the group with commercial biostimulant.

Starch is the main source of energy in wheat grain. Generally, it ranges from 60% to 70% of grain mass [30]. Since wheat is the second most important energy source for the human population, starch content in the grains was determined in the present study. The content of starch in grains from all the tested group was in the mentioned range and was comparable in all groups.

It was shown that the tested biostimulants, i.e., hydrolysates, as well as commercial product increased the average weight of 1000 grains when compared with the control group: by ~1.5% for Asahi SL; ~2% for AminoHort (1.25 L/ha); ~2.2% for AminoPrim; and ~2.4% for AminoHort (1.0 L/ha). However, these differences were not statistically significant.

The quality of the grains was also determined by their mineral composition. Considering multi-elemental composition of the tested amino acid hydrolysates (AminoPrim and AminoHort: minor amounts of macroelements; N_total_ 2.8% and 3.5%, respectively; P_2_O_5_ 0.8% and 1.1%; K_2_O 3.9% and 4.5%; and microelements), it can be suggested that their function was to increase the nutrient uptake by plants from the environment. This is in agreement with the literature data: biostimulants act by increasing plant mineral uptake and by improving the nutrients use efficiency [1,3,31]. The level of selected macro- and micronutrients in wheat grains is shown in Table 7, respectively. The statistical analysis revealed significant differences in the case of copper. For the control object, the content of this element was 4.58 mg/kg, while for objects with AminoPrim (1.0 L/ha) and AminoHort at 1.0 and 1.25 L/ha, it was 6.01, 6.67, and 6.87 mg/kg (increased by 31%, 46% and 50%, respectively). When compared to the commercial biostimulant, the content of copper increased by 15%, 28% and 32%, respectively. For the other macro- and micronutrients, differences were not statistically significant; however, the use of preparations based on amino acids had a positive impact on the increase of these elements in wheat grain. The largest increase was found for sodium, calcium and molybdenum. The content of sodium increased in grains of plants treated with AminoPrim (1.0 L/ha) and AminoHort (1.0 and 1.25 L/ha) by 36%, 43% and 47%; calcium by 4.3%, 5.3% and 7.9%; and molybdenum by 4.0%, 15% and 16%, respectively. Biostimulants with amino acids also significantly modified sodium and calcium content in the grain when compared to the commercial plant growth stimulator. There was an increase of these elements by 35%, 42%, and 46%, and 3.6%, 4.6%, and 7.2%, respectively, for Na and Ca. The increased efficiency of the tested biostimulants can be associated not only with the presence of micro- and macronutrients in hydrolysates, but also amino acids obtained by hydrolysis of keratin material, i.e., feathers. Amino acids are good chelating agents and act as carriers of micronutrients contained in the preparations or soil [12,15,18,19]. The effect of biostimulants of plant growth results from the synergism of many components at different concentrations [31].

Sustainable agriculture requires using not only effective mineral fertilizers containing macro- and microelements, but also plant growth biostimulants which are a rich source of biologically active compounds. These very important formulations allow achieving significant increases in the quality and quantity of yield, as well as improve the health of plants. Moreover, these preparations improve the efficiency of fertilizer nutrients uptake. Protein hydrolysates are an important group of plant growth biostimulants based on a mixture of peptides and amino acids [9]. The use of amino acids is most often recommended under critical conditions of plant growth: after transplantation, in the flowering period and during climatic stresses (night frosts and drought) or plant diseases [14]. In fertilizers, amino acids form organic connections with minerals (amino acid chelates), which increase the availability of nutrients by plants [15,18,19].

Additionally, in the method of production of biostimulants with amino acids, feathers were used as a keratin material. This approach allows the recovery of nutrients and is one of the recommended strategy for dealing with waste according to the Directive 2008/98/EC, 2008 [32]. Poultry feathers are a rich source of nitrogen (ca. 15%), acting as the main growth factor and sulfur (>2.0%), often mentioned as a deficient component in agricultural soils. Feathers are also a source of desired micronutrients in plant cultivation, particularly iron and zinc. The content of toxic metals is at an extremely low level [33], far ahead of the limit values set by the requirements for organic–mineral fertilizers by the Polish Regulation of Minister of Agriculture and Rural Development of 18 June 2008 on the implementing some provisions of the Fertilizers and Fertilizing Act. Journal of Laws, No. 119 Pos. 765, 2008.

## 3. Materials and Methods

### 3.1. Tested Preparations Based on Amino Acids

In the study, the plant growth stimulants (AminoPrim and AminoHort), which were produced according to the technology elaborated at Wrocław University of Science and Technology (Poland) and currently produced by INTERMAG Co. (Olkusz, Poland) were used. The basic component of both formulations is a liquid containing highly concentrated mixture of amino acids and short peptides, obtained in the hydrolysis (H_2_SO_4_/H_3_PO_4_; POCh S.A., Gliwice, Poland) of keratin material (feathers) and then enriched with selected nutrients. The correction of micronutrient composition was made by their introduction in the form of water soluble inorganic salts, allowed for the use as components of fertilizers with microelements according to the Regulation EC No. 2003/2003 [34] (H_3_BO_3_; CuSO_4_·5H_2_O; FeSO_4_·7H_2_O; MnSO_4_·1H_2_O; (NH_4_)_6_Mo_7_O_24_·4H_2_O; ZnSO_4_·7H_2_O; POCh S.A., Gliwice, Poland). The process of hydrolysis was carried out under controlled conditions for 6–8 h in temperature of 85 °C. The next step was regulation of pH of mixture to the value 6 by adding KOH. Finally, the filtration process allowed separating insoluble parts in product. The chemical composition of the preparations based on amino acid is given in Table 2. The method of production is protected by patents [35,36,37,38].

### 3.2. Field Experiment

The field experiment was carried out by Agreco Co. (Poland) during 2012–2013 (planting day of wheat was 20 October 2012; harvest was on 12 August 2013). The test was carried out on winter wheat (*Triticum aestivum* L.) variety Tacitus. Plants grown in soil composed mainly of light sandy loam. The quality class was III b. The soil was characterized by pH value of 7.4 (pH in 1 M KCl) and the organic matter content of 2.2%. The previous crop before wheat was sugar beet. The experiment was set up in the randomized block design in the quadruplicates. The area of plots for sowing was 24 m^2^ (3.2 × 7.5 m).

The study included evaluation of the properties of biostimulants based on amino acids—AminoPrim at a dose 1.0 L/ha and AminoHort at doses 1.0 and 1.25 L/ha. The preparations were compared to the commercial plant growth stimulator Asahi SL (Asahi Kasei, Tokyo, Japan) applied at a dose recommended by the manufacturer (0.6 L/ha) and the control group (without biostimulant). Asahi was chosen as a reference material since it is registered as a biostimulant of plant growth on the Polish market by the Ministry of Agriculture and Rural Development in Poland. According to the recommendations of the producer, 0.6 L of Asahi should be dissolved in 300 L of water. The selection of the dose of AminoPrim and AminoHort was based on our previous pot experiments on mustard, where these preparations were applied by foliar spraying at doses corresponding to large-scale fertilization of 1.5 L, 3.0 L and 6.0 L per hectare. The best results considering fresh mass and chlorophyll content were obtained for the dose 1.5 L/ha [39]. Therefore, in the present study, 1 L and the increased dose of obtained hydrolysate, 1.25 L, were dissolved in 300 L of water and applied foliarly on 1 ha. The preparations were used twice: 23 April 2013 and 30 May 2013 (Table 8).

The standard fertilization (NPK) was not applied. During 23 April 2013–10 June 2013 systematic applications of plant protection products were carried out. Before sowing wheat, disking and harrowing were done. Weather conditions (average temperature in °C and total rainfall in mm) in these months were as follows: April: 8.7 °C and 41.4 mm; May: 15.0 °C and 95.2 mm; June 17.2 °C and 158 mm; July: 20.5 °C and 29.0 mm; and August: 20.1 °C and 61.0 mm.

In the experiment, such parameters as plant vigor, leaf color, selectivity (visual evaluation of phytotoxicity), plant height, tillers (number of first, second and third tillers), ear number per m^2^, lodging (area and intensity) and grain yield quantity were determined [40]. The methods of observations and measurements are given in Table 9.

### 3.3. Laboratory Experiments

After harvesting of winter wheat, the weight of 1000 grains according to PN-R-74017 (PN-EN ISO 520:2011, 2001) [41]; the ash content in the grain according to ISO 2171 (PN-EN ISO 2171:2010, 2010) [42]; the content of protein, wet gluten and starch; and Zeleny sedimentation index using the near-infrared (NIR) method (InfratecTM 1241 Grain Analyzer, Foss, Denmark) were determined.

The grain of winter wheat was also subjected to multi-elemental analysis (macro- and micronutrients) using ICP-OES technique (Inductively Coupled Plasma–Optical Emission Spectrometry) (Vista MPX, Varian Inc., Palo Alto, CA, USA). Before the analysis, the samples of grain were crushed and then (0.5 g) subjected to digestion with high purity nitric acid (69%, 5 mL) (Suprapur Merck KGaA, Darmstadt, Germany) in Teflon bombs in the microwave oven (Milestone S.r.l. – START D, Sorisole, Italy). After digestion process, the samples were diluted with doubly deionized water (Millipore Simplicity system, Merck KGaA, Darmstadt, Germany) to weight of 50 g. The study was performed in the Chemical Laboratory of the Multielemental Analyses (Wrocław University of Science and Technology, Poland) accredited by the Polish Centre for Accreditation (No. AB 696), using standard analytical procedures of the laboratory. Nitrogen was determined by the Kjeldahl method using digester (K-424/435, BÜCHI, Flawil, Switzerland) and distillation unit (B-324, BÜCHI, Switzerland). To determine the amino acid composition, chromatographic analysis was carried out. The analysis was performed on amino acid analyzer (AAA 400 Ingos; Prague, Czech Republic).

### 3.4. Statistical Analysis

The statistical analysis of results (ANOVA analysis) was conducted with Tukey’s test for the reasonable significant difference (RIR) at the significance level *p* = 0.01 and 0.05. The calculations were made using the *STATISTICA* program version 10 (Cracow, Poland).

## 4. Conclusions

In the present paper, the results of field trials on winter wheat are presented. As tested products, two formulations (AminoPrim and AminoHort) were examined. Their active component is a liquid mixture of amino acids and short peptides, obtained by the hydrolysis (H_2_SO_4_/H_3_PO_4_) of keratin material (feathers), and enriched with some nutrients. The tested formulations combined the properties of biostimulant of plant growth and micronutrient fertilizer. The study has shown a positive effect of the use of preparations with amino acids in the cultivation of winter wheat. Neither phytotoxic symptoms nor lodging of plants were observed. The plant vigor and leaf color of wheat were at the optimum level. There was an increase of the yield in all the tested groups when compared with the control group, but the best results were obtained for AminoHort at dose 1.25 L/ha (higher by 11%). Considering the technological characteristics of grain, it was found that the ash content was the highest in the group treated with AminoHort at a dose 1.0 L/ha (increased by ~8%, compared with the control group), the Zeleny sedimentation index and protein content was the highest for AminoHort at a dose 1.25 L/ha (increased by ~10% and ~3%, respectively, compared to the control group). The average weight of 1000 grains, starch content and wet gluten were comparable in all tested groups. The use of formulations based on amino acids contributed to the increase of nutrients in the grain, in particular macronutrients such as Na and Ca, and micronutrient such as Cu and Mo. Multielemental analysis showed higher contents of these elements when compared to the commercial plant growth stimulator (Asahi SL). For further research, the formulation AminoHort is recommended, however, field trials will be performed for higher doses of the tested biostimulant and with the application of the standard fertilization. Additionally, it is important to conduct more experiments to understand the action mechanisms of the biostimulants on plants.

## Figures and Tables

**Table 1:** Fundamental functions of the amino acids in plants [14].

Function	Amino Acid
Anti-stress agent	Hyp, Pro
Chelating agent	Cys, Glu, Gly, His, Lys
Cold weather resistance	Ala, Arg
Generative development of plants and improvement of the plant pollen fertility	Hyp, Pro
Growth stimulator	Glu
Precursor of auxin	Ser, Trp, Val
Precursor of chlorophyll	Gly
Precursor of polyamines: necessary to start the cell division	Arg
Precursor to the formation of lignin and woody tissues	Phe
Regulation of the water balance	Hyp, Pro, Ser
Reserve of organic nitrogen necessary for the synthesis of other amino acids and proteins	Glu
Stimulation of the chlorophyll synthesis	Ala, Lys, Ser
Stimulation of the ethylene synthesis	Met
Stimulation of the germination	Asp, Glu, Lys, Met, Phe, Thr
Stimulation of the hormone metabolism	Ala
Stimulation of the resistance mechanism to viruses	Ala

**Table 2:** The chemical composition of the tested preparations.

Component	AminoPrim	AminoHort
**Macroelement (%)**		
N_total_	2.8	3.5
P_2_O_5_	0.8	1.1
K_2_O	3.9	4.5
CaO	0.2	0.2
MgO	2.0	2.0
SO_3_	6.8	9.2
**Microelement (%)**		
B	0.02	0.2
Cu	0.05	0.1
Fe	0.1	1.0
Mn	0.05	0.3
Mo	0.001	0.005
Zn	0.05	0.5
**Amino acid (%)**		
Asp	1.4	1.7
Thr	0.5	0.7
Ser	1.5	1.9
Glu	1.6	2.3
Pro	1.7	2.0
Gly	1.4	1.9
Ala	0.8	1.1
Val	1.2	1.5
Ile	0.6	0.8
Leu	1.4	1.7
Tyr	0.5	0.6
Phe	0.7	0.9
His	0.2	0.4
Lys	0.2	0.5
Arg	1.0	1.3
Cys	0.5	0.8
Met	0.04	0.2
Trp	0.01	0.01

**Table 3:** Plant height of the tested winter wheat.

Variant	Dose (L/ha)	Application time/Crop growth stage (BBCH scale)	
		30 May 2013/43–45	25 June 2013/75
		Average plant height (cm) (*n* = 4) ± SD	
Control	-	35.6 ± 0.7	43.7 ± 0.9
AminoPrim	1.0	35.9 ± 0.9	43.4 ± 1.0
AminoHort	1.0	36.6 ± 1.6	44.3 ± 1.4
	1.25	35.7 ± 0.9	43.3 ± 0.9
Asahi SL	0.6	36.8 ± 1.6	43.1 ± 0.6

SD—standard deviation.

**Table 4:** Tillering evaluation of the tested winter wheat.

Variant	Dose (L/ha)	Application time/Crop growth stage (BBCH scale)		
		25 June 2013/75		
		The average tillers number (*n* = 25 plants per plot) ± SD		
		1	2	3
Control	-	1.8 ± 0.1	1.3 ± 0.1	0.4 ± 0.1
AminoPrim	1.0	1.9 ± 0.1	1.3 ± 0.1	0.6 ± 0.1
AminoHort	1.0	1.9 ± 0.1	1.3 ± 0.1	0.7 ± 0.1
	1.25	1.8 ± 0.1	1.3 ± 0.2	0.7 ± 0.2
Asahi SL	0.6	1.8 ± 0.1	1.6 ± 0.2	0.5 ± 0.1

**Table 5:** The average ear number and grain yield of the tested winter wheat.

Variant	Dose (L/ha)	Application Time/Crop Growth Stage (BBCH scale)	
		20 August 2013/89	
		Average Ear Number/m^2^ (*n* = 4) ± SD	Average Grain Yield (t/ha) (*n* = 4) ± SD
Control	-	375 ± 39	4.98 ± 0.49
AminoPrim	1.0	390 ± 53	5.25 ± 0.92
AminoHort	1.0	425 ± 76	4.64 ± 1.07
	1.25	392 ± 46	5.55 ± 0.48
Asahi SL	0.6	440 ± 28	5.08 ± 0.58

**Table 6:** Technological characteristics of the winter wheat grain.

Variant	Dose (L/ha)	Moisture (%)	1000 grains weight (g)	Ash (% d.m.)	Protein (% d.m.)	Starch (% d.m.)	Wet gluten (%)	Zeleny sedimentation index (mL)
Control	-	14.6 ± 1.13	46.1 ± 0.11	1.53 ± 0.03 ^AaBb^	12.8 ± 0.98	68.2 ± 1.61	31.0 ± 4.40	37.3 ± 7.41
AminoPrim	1.0	14.5 ± 0.25	47.1 ± 0.11	1.62 ± 0.05 ^a^	13.1 ± 1.77	68.0 ± 1.23	31.5 ± 5.19	38.2 ± 15.4
AminoHort	1.0	14.6 ± 0.47	47.2 ± 0.07	1.65 ± 0.04 ^A^	13.1 ± 1.42	68.4 ± 1.32	30.9 ± 4.06	40.7 ± 13.8
	1.25	14.7 ± 0.42	47.0 ± 0.06	1.64 ± 0.03 ^B^	13.2 ± 1.21	68.2 ± 1.34	31.5 ± 3.95	40.9 ± 11.3
Asahi SL	0.6	14.4 ± 0.70	46.8 ± 0.06	1.62 ± 0.03 ^b^	12.9 ± 0.84	68.0 ± 0.68	30.0 ± 2.82	38.4 ± 12.7

Average ± SD; d.m., dry mass; ^A,B^ values designated with the same letters in a single column indicate high significant differences according to Tukey’s test (*p* ≤ 0.01); ^a,b^ values designated with the same letters in a single column indicate significant differences according to Tukey’s test (*p* ≤ 0.05).

**Table 7:** The average macro- and micronutrient content in a winter wheat grains.

**Variant**	**Dose (L/ha)**	**Average Content of Macroelements (g/kg d.m.; *n* = 4) ± SD**					
		**Ca**	**K**	**Mg**	**Na**	**P**	**S**
Control	-	0.303 ± 0.03	4.14 ± 0.34	1.40 ± 0.04	0.200 ± 0.07	4.67 ± 0.11	1.63 ± 0.06
AminoPrim	1.0	0.316 ± 0.04	4.22 ± 0.30	1.46 ± 0.04	0.271 ± 0.08	4.84 ± 0.10	1.68 ± 0.08
AminoHort	1.0	0.319 ± 0.02	4.37 ± 0.34	1.46 ± 0.05	0.286 ± 0.02	4.84 ± 0.02	1.72 ± 0.06
	1.25	0.327 ± 0.01	4.32 ± 0.32	1.44 ± 0.06	0.293 ± 0.02	4.93 ± 0.14	1.70 ± 0.13
Asahi SL	0.6	0.305 ± 0.02	4.08 ± 0.21	1.42 ± 0.04	0.201 ± 0.03	4.65 ± 0.08	1.65 ± 0.09
**Variant**	**Dose (L/ha)**	**Average Content of Microelements (mg/kg d.m.; *n* = 4) ± SD**					
		**B**	**Cu**	**Fe**	**Mn**	**Mo**	**Zn**
Control	-	< LOD*	4.58 ± 0.42 ^AaB^	29.3 ± 3.42	23.7 ± 1.13	1.52 ± 0.15	50.4 ± 5.62
AminoPrim	1.0	< LOD*	6.01 ± 0.69 ^a^	29.6 ± 4.15	23.7 ± 1.68	1.58 ± 0.10	51.7 ± 5.12
AminoHort	1.0	< LOD*	6.67 ± 0.35 ^Ab^	31.3 ± 3.98	24.7 ± 2.05	1.75 ± 0.25	53.1 ± 4.93
	1.25	< LOD*	6.87 ± 0.45 ^BC^	32.3 ± 4.84	25.2 ± 1.80	1.76 ± 0.16	54.6 ± 5.20
Asahi SL	0.6	< LOD*	5.22 ± 0.28 ^bC^	32.4 ± 3.75	23.6 ± 2.36	1.60 ± 0.12	50.3 ± 5.37

^A,B,C^ values designated with the same letters in a single column indicate high significant differences according to Tukey’s test (*p* ≤ 0.01); ^a,b^ values designated with the same letters in a single column indicate significant differences according to Tukey’s test (*p* ≤ 0.05); * LOD_B_ < 0.0315 mg/kg (below limit of detection).

**Table 8:** Doses of tested and reference products.

Treatment No.	Product	Dose (L/ha)	Application No. *
1	Control	-	-
2	AminoPrim	1.0	I, II
3	AminoHort	1.0	I, II
4		1.25	I, II
5	Asahi SL	0.6	I, II

* Application time/Crop growth stage (BBCH scale): I, 23 April 2013/29–31; II, 30 May 2013/43–45

**Table 9:** Assessment methods.

Assessment time *	Date	Crop growth stage (BBCH scale)	Assessment type
14 days after application I	7 May 2013	32–34	Plant vigor on a 0–10 scale: 0—plant death, 5—optimum vigor (untreated), 10—most vigorous plants per plot Leaf color on a 0–10 linear scale: 0—no color, 5—optimal color (untreated), 10—most green color per plot Selectivity: visual evaluation of phytotoxicity per plot) (%)
30 days after application I	23 May 2013	39–41	
Before application II	30 May 2013	43–45	Plant vigor on a 0–10 scale Leaf color on a 0–10 linear scale Selectivity (%) Plant height (cm)
Medium milk 26 days after application II	25 June 2013	75	Plant vigor on a 0–10 scale Leaf color on a 0–10 linear scale Selectivity (%) Plant height (cm) Tilleres: number of first, second and third tillers (25 plants per plot)
Before harvest	20 August 2013	89	Ear number per m^2^ Lodging: area lodged (%), intensity of lodging
Harvest	23 August 2013	89	Grain yield quantity (kg/plot) Grain moisture (%) Grain yield quantity based on a standard 14% moisture (t/ha)

* Application time/Crop growth stage (BBCH scale): I, 23 April 2013/29–31; II, 30 May 2013/43–45
